# The influence of memory on the speech-to-song illusion

**DOI:** 10.3758/s13421-021-01269-9

**Published:** 2022-01-26

**Authors:** Lauren E. Soehlke, Ashwini Kamat, Nichol Castro, Michael S. Vitevitch

**Affiliations:** 1 Department of Psychology, University of Kansas, Lawrence, KS USA; 2 University at Buffalo, Buffalo, NY USA

**Keywords:** Memory, Music cognition, Perception, Psycholinguistics

## Abstract

In the speech-to-song illusion a spoken phrase is presented repeatedly and begins to sound as if it is being sung. Anecdotal reports suggest that subsequent presentations of a previously heard phrase enhance the illusion, even if several hours or days have elapsed between presentations. In Experiment 1, we examined in a controlled laboratory setting whether memory traces for a previously heard phrase would influence song-like ratings to a subsequent presentation of that phrase. The results showed that word lists that were played several times throughout the experimental session were rated as being more song-like at the end of the experiment than word lists that were played only once in the experimental session. In Experiment 2, we examined if the memory traces that influenced the speech-to-song illusion were abstract in nature or exemplar-based by playing some word lists several times during the experiment in the same voice and playing other word lists several times during the experiment but in different voices. The results showed that word lists played in the same voice were rated as more song-like at the end of the experiment than word lists played in different voices. Many previous studies have examined how various aspects of the stimulus itself influences the perception of the speech-to-song illusion. The results of the present experiments demonstrate that memory traces of the stimulus also influence the speech-to-song illusion.

In the auditory illusion known as the speech-to-song illusion, a spoken phrase is presented repeatedly and begins to sound as if it is being sung instead of spoken. Although experimental musicians were using this phenomenon to artistic effect several decades ago (e.g., “It’s Gonna Rain,” by Steve Reich, 1965), experimental psychologists did not study this phenomenon until Diana Deutsch observed the illusion while recording descriptions of other auditory illusions (Deutsch, 1995, 2003). Since the initial scientific report of the speech-to-song illusion (Deutsch et al., 2011), the illusion has been replicated with other phrases in English (Rowland et al., 2019), as well as in German (Falk & Rathcke, 2010) and Mandarin (Zhang, 2011), demonstrating the universality of the illusion.

Many studies have examined various factors of the stimulus—such as pitch, rhythm, and other acoustic features—that increase or decrease the probability of evoking the speech-to-song illusion, or that increase or decrease the strength of the illusion as measured by the song ratings of the stimuli (e.g., Falk & Rathcke, 2010; Falk et al., 2014; Groenveld, Burgoyne, & Sadakata, 2020; Jaisin et al., 2016; Margulis et al., 2015; Rowland et al., 2019; Tierney et al., 2018). In the present set of experiments, however, we wanted to examine how *memory* might influence this auditory illusion. Given the obvious role that the physical stimulus plays in eliciting an illusion it might seem illogical to examine how memory might affect a perceptual illusion. However, several converging pieces of information motivated us to examine how memory might affect this particular perceptual illusion.

First, there are anecdotal reports about the speech-to-song illusion being enhanced when the stimulus is subsequently presented. That is, once people hear the phrase “Sometimes behave so strangely,” they almost immediately perceive it as being song-like on subsequent hearings of that phrase, even if several hours or days have passed, suggesting that memories of the previously experienced stimulus may influence the perception of this illusion.

Second, work on optical illusions by Scocchia et al. (2014) found that the learned experience (i.e., memory) of observers influenced the perception of certain optical illusions. In the case of the speech-to-song illusion, Vanden Bosch der Nederlanden et al. (2015) found that everyday musical experience (i.e., memory) is sufficient to evoke the speech-to-song illusion in listeners. Thus, there is some empirical support that memory might affect perception of various illusions.

Additional empirical evidence that memory might affect perception of the speech-to-song illusion comes from Gronveld et al. (2020), who presented listeners with phrases that were demonstrated in a previous study to not elicit the speech-to-song illusion (Cornelissen et al., 2016). Gronveld et al. manipulated the contour of the fundamental frequency (F0) of the speech segments (F0 contour manipulations of 0%, 30%, 60%, and 90%) to make the stimuli increasingly more likely to elicit the speech-to-song illusion and be rated more song-like.

Listeners in the Gronveld et al. (2020) study were then presented repeatedly with the speech samples in three conditions: increasing from not-song-like to song-like (the stimulus with 0% contour manipulation, then the same stimulus with 30% contour manipulation, etc.), decreasing from song-like to not-song-like (the stimulus with 90% contour manipulation, then the same stimulus with 60% contour manipulation, etc.), or with the contour manipulations presented in random order. They found only in the decreasing condition of F0 contour manipulations (shifting from song-like to not-song-like) that listeners continued to give higher overall song-like ratings in the experimental session, which they interpreted as evidence “that it is hard to ‘unhear’ the illusion once a speech segment has been perceived as song” (Gronveld et al., 2020, p. 1451). Said another way, the initial memory trace of a canonical, song-like stimulus influenced (i.e., increased) song ratings for subsequent presentations of the phrase even when the auditory signal in the subsequent presentations was less than optimal for eliciting the speech-to-song illusion.

The final converging piece of information that motivated us to examine how memory might affect the speech-to-song illusion is that rhythmic groupings of auditory stimuli are known to enhance the serial recall of word lists (Hartley et al., 2016; Ryan, 1969), and to enhance (to a lesser extent compared with acoustic features) long-term memory for music (Hébert & Peretz, 1997). Numerous studies have identified cognitive and neurological connections between speech and music (e.g., Patel et al., 1998; Peretz et al., 2015), but the emphasis on rhythm in the present case is important because one account of the speech-to-song illusion appeals to rhythm as a contributor to the speech-to-song illusion. (Note that other accounts of the speech-to-song illusion will be discussed later.) This rhythm-based account of the speech-to-song illusion draws on the mechanisms in a language processing model called node structure theory (NST;MacKay, 1987 ; MacKay et al., 1993).

Recent findings from Castro et al. (2018; see also Mullin et al., 2021; Vitevitch et al., 2020) indicate that the mechanisms in NST (MacKay, 1987; MacKay et al., 1993)—priming, activation, and satiation—may explain how speech can be perceived as being song-like after several presentations. In NST, nodes are used to represent phonemes, syllables, words, and other types of linguistic information. Links connect nodes such that phoneme nodes connect to syllable nodes, syllable nodes connect to lexical nodes, and so forth (see Fig. 1). During speech perception incoming acoustic-phonetic information *primes* (similar to spreading activation in other models; e.g., Collins & Loftus, 1975) phonological nodes, based on the extent to which the nodes match the input. When a node accumulates enough priming to surpass an activation threshold the node is *activated*, bringing to conscious awareness the information represented by that node.

**Fig. 1:**
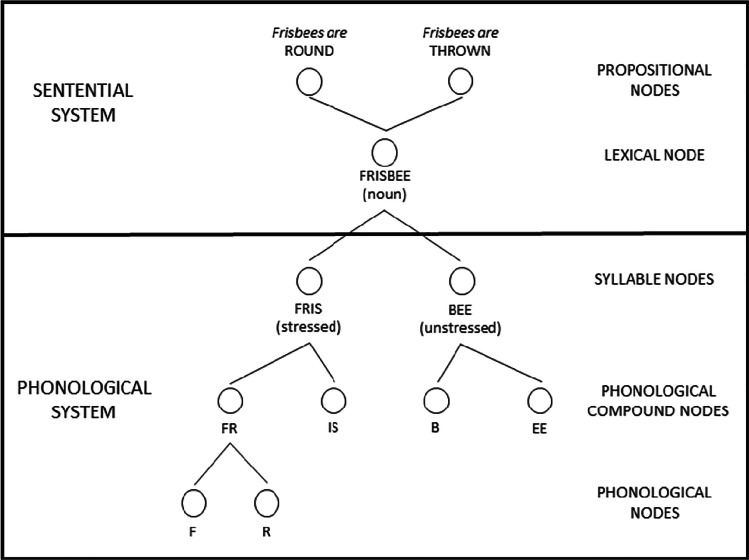
Nodes representing various types of linguistic information for the word *Frisbee*. Additional higher-level and lower-level nodes described in node structure theory have been omitted to simplify the image. For ease of presentation, we use orthographic symbols rather than symbols from the International Phonetic Alphabet (IPA) to represent the phonological sounds found in the syllables and words represented in the figure. Adapted from Fig. 1 in MacKay (1987)

Presentation of the phrase initially primes and activates lexical nodes associated with the words in that phrase, bringing to conscious awareness a speech-like percept. With repeated activation of the same lexical nodes, *satiation* occurs, resulting in the lexical nodes being temporarily unable to accumulate priming and be activated, and in the loss of the initial speech percept. Even though the lexical nodes are in a state of satiation, additional presentations of the stimulus continue to prime the syllable nodes. Because syllables—widely recognized as a unit of rhythmic structure in speech (e.g., Cutler, 1991; Fujii & Wan, 2014; Jackendoff, 2009; Ramus et al., 1999)—continue to receive priming, the syllable nodes make salient the rhythmic pattern in the repeated phrase, producing a song-like percept.

Note that only nodes that have been activated—such as the lexical nodes in the account of the speech-to-song illusion provided above—experience satiation (MacKay, 1987). During typical speech perception it is sufficient to prime, but not fully activate nodes in the phonological system, including syllable nodes and nodes representing individual phonemes. The priming, but not the activation, of the phoneme and syllable nodes allows priming to be transmitted to and for the activation of the lexical nodes, resulting in the listener perceiving words rather than sequences of phonemes when listening to speech. In the case of the speech-to-song illusion, satiation of the activated lexical nodes results in the loss of the speech percept, but the continued priming of the rhythmic structure of speech encoded in the syllable nodes (which are not activated, and therefore do not satiate) results in the percept shifting to something more music-like than speech.

Given the anecdotal and empirical evidence of previous exposure to a phrase influencing the subsequent illusory perception of it (e.g., Gronveld et al., 2020), evidence that learned experience can influence the perception of certain optical illusions (e.g., Scocchia et al., 2014), the enhanced memory for rhythmic groupings of word lists (Ryan, 1969), and a rhythm-based account of the speech-to-song illusion based on the mechanisms of the language model NST (e.g., Castro et al., 2018), we sought in the present studies to examine how memory might influence the speech-to-song illusion. In the two experiments reported here we used methods typically employed in the study of spoken word recognition to examine how memory for a previously heard phrase might influence the subsequent illusory perception of it. Approaching the speech-to-song illusion from the “speech” perspective contrasts with the more typical approach of research on the speech-to-song illusion, which more often has been from the “song” perspective of music cognition (e.g., Deutsch et al., 2011; Margulis & Simchy-Gross, 2016).

## Experiment 1

To capture in the laboratory the influence that memories for a previously experienced stimulus may have on the speech-to-song illusion, we presented listeners with lists of words that are known to evoke the speech-to-song illusion—namely, the stimuli from Experiment 1of Castro et al. (2018). Some of the lists were presented multiple times during the experimental session, whereas other lists were presented only once during the experimental session. We chose to use these lists not only because they have been shown to elicit the speech-to-song illusion but also because the concatenation of four words to form a list minimizes the influence of phrasal prosody, syntax, and so on, allowing us to focus on the research question at hand.

We also used the same task used in Castro et al. (2018) and in many other studies of the speech-to-song illusion. Participants listened to 10 repetitions of each stimulus. At the end of the 10 repetitions, the participants provided a rating on a 5-point Likert scale, with 1 corresponding to “sounds like speech” and 5 corresponding to “sounds like song.” Higher ratings on the scale indicate experiencing a song-like percept, whereas lower ratings on the scale indicate perceiving the stimulus as sounding more like normal speech. If the anecdotal reports of a previously heard stimulus enhancing in some way the experience of the illusion when the stimulus is heard subsequently, then we expect in our laboratory-based analogue to observe increases in song-likeness ratings for the word lists that are presented again during the experimental session compared to the novel word lists.

### Methods

#### Participants

Based on the effect sizes, statistical power, and sample sizes of previous speech to song experiments (e.g., Castro et al., 2018), we established the stopping rule of collecting data from 40 participants or the end of the semester occurs, resulting in 34 native English speakers being recruited from a pool of students enrolled in Introductory Psychology at the University of Kansas by the end of the semester. Participants received partial credit toward the completion of the course for their participation. All were native English speakers, and none reported a hearing or speech disorder. Written informed consent was obtained before participating in the experiment, and this experiment was approved by the Institutional Review Board at the University of Kansas.

#### Stimuli

The 14 lists of four words used in Experiment 1 of Castro et al. (2018) were used in the present experiment. As described in Castro et al., the 56 bisyllabic words were originally used in Vitevitch et al. (2008). Each word was recorded in isolation by a female speaker and concatenated to form the lists of four words. Approximately 50 ms of silence occurred between the onset of each word in the list and between the onset of each repetition of the four words in each list.

As further described in Castro et al. (2018), the words had a strong-weak stress pattern, the same phonemes occurred in each condition equivalent numbers of times, and the number of fricatives that appeared in each condition was also equivalent. Some lexical variables typically examined in psycholinguistic studies were controlled, including word frequency, neighborhood frequency, length of the word as measured by the number of phonemes, and uniqueness points. Finally, as described in Castro et al., the words were also equivalent in duration, and in the minimum and maximum pitch values. The number of linguistic and acoustic features that were comparable across the lists suggests that the rhythmic structure across the lists was also comparable.

Seven of the lists contained four words with dense phonological neighborhoods (i.e., each word had many similar sounding words), and the remaining lists contained four words with sparse phonological neighborhoods (i.e., each word had few similar sounding words; Luce & Pisoni, 1998; Vitevitch & Luce, 2016). As described in Castro et al. (2018), none of the words in a list were phonological neighbors of another word in the list. Our interest in the present experiment was not on the variable of neighborhood density (as it was in Experiment 1 of Castro et al., 2018); we simply wished in the present case to use stimuli that were known to evoke the speech-to-song illusion as demonstrated in Castro et al. (2018).

See Table 1 for a visual representation of how the lists were presented to participants. Twelve of the lists were presented only once during the experiment (referred to as the *novel* condition). Two lists were presented a total of 4 times during the experiment (referred to as the *familiar* condition), resulting in a total of 20 trials during the experiment. These 20 trials were separated into four blocks, which contained five word lists that were randomly presented in a different order for each participant. There was no time delay between the presentation of each block; we simply use the term “block” to facilitate description of how the stimuli were presented during the experimental session.

**Table 1:** A visual representation of how the stimuli (from List B as designated in the Appendix) were presented in the experimental session

	Block 1	Block 2	Block 3	Block 4
Novel	List D6	List D2	List D1	List D4
	List S2	List D7	List S1	List D5
	List S6	List S7	List S5	List S4
Familiar	List D3	List D3	List D3	List D3
	List S3	List S3	List S3	List S3

*Note.* Each participant received the same five word lists with-in each block, but presentation order within each block was in a different randomized order for each participant. D = list of dense words as designated in the Appendix. S = list of sparse words as designated in the Appendix

Three of the lists in each block were in the *novel* condition, meaning that they were presented only once during the experiment. Two of the lists in each block were in the *familiar* condition, meaning that they were presented in all four blocks of the experiment (and in a randomized order in each block). (See the Appendix for the words in each list and for which lists were in the *novel* and *familiar* conditions.) Eighteen participants received the first pseudo-randomized presentation order (designated List A in the Appendix), and 16 participants received the second pseudo-randomized presentation order (designated List B in the Appendix).

#### Procedure

Participants were tested individually. Each participant was seated in front of an iMac computer running PsyScope 1.2.2 (Cohen et al., 1993). This program-controlled stimulus presentation and collected responses.

The word “READY” appeared on the computer screen for 500 ms at the start of each trial. Participants then heard one of the word lists repeated 10 times through a set of Beyerdynamic DT 100 headphones at a comfortable listening level. To be clear, the identical list of four words was repeated; there were not different tokens of each word, or any variation in the acoustics across the 10 repetitions. After the repetitions, participants were instructed to use the number pad on the keyboard to rate the list on a scale of 1 (*sounded more like speech*) to 5 (*sounded more like song*). Participants were allowed as much time as they needed to respond. In total, the experiment lasted approximately 10 to 15 minutes.

We present here an example of how a few trails proceeded in the experiment using the word lists and ordering depicted in Table 1. After the word “READY” appeared on the computer screen, the list containing the words would be presented 10 times (e.g., *dairy, meter, body, lighter; dairy, meter, body, lighter; dairy, meter, body, lighter; dairy, meter, body, lighter; dairy, meter, body, lighter; dairy, meter, body, lighter; dairy, meter, body, lighter; dairy, meter, body, lighter; dairy, meter, body, lighter; dairy, meter, body, lighter*). After the 10th repetition, the participant would be prompted to rate on the 5-point scale the song-likeness of the word list.

Once the rating was entered, the word “READY” appeared on the computer screen, and the next list would be presented 10 times (e.g., *lawyer, mother, button, barrel; lawyer, mother, button, barrel; lawyer, mother, button, barrel; lawyer, mother, button, barrel; lawyer, mother, button, barrel; lawyer, mother, button, barrel; lawyer, mother, button, barrel; lawyer, mother, button, barrel; lawyer, mother, button, barrel; lawyer, mother, button, barrel*) with the song-likeness rating being made after the 10th repetition of the word list. The remaining trials in the experiment proceeded in a similar manner.

### Results

**Fig. 2:**
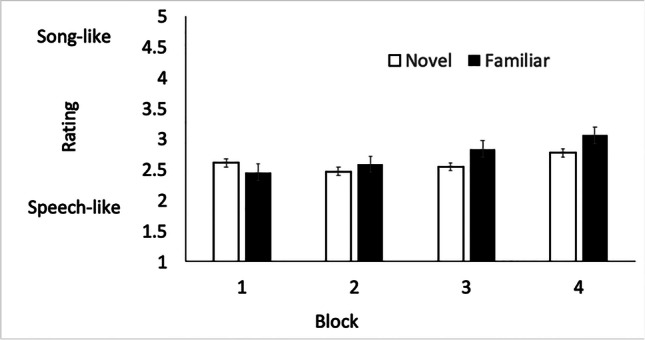
Song-like ratings (and standard error of the mean) for the *novel* and *familiar* word lists across the four presentation blocks in Experiment 1

A two-way (Blocks × Word lists) repeated-measures analysis of variance (ANOVA) was used to analyze the data (see Fig. 2). There were four presentation blocks during the experimental session, and word lists were either *novel* or *familiar*. The main effect of word list was not significant, with *novel* word lists (*M* = 2.74, *SD* = .99) being rated overall about the same as *familiar* word lists (*M* = 2.60, *SD* = .86), *F*(1, 33) = 1.571, *p* = .219. The main effect of presentation blocks was significant, with the ratings indicating that overall the stimuli became more song-like across the experimental session: Block 1 (*M* = 2.53, *SD* = .77), Block 2 (*M* = 2.53, *SD* = .87), Block 3 (*M* = 2.69, *SD* = 1.01), Block 4 (*M* = 2.92, *SD* = 1.04), *F*(3, 99) = 3.65, *p* = .015.

Crucially, the interaction between blocks and word list was statistically significant, suggesting that over time the repeated *familiar* word lists were rated as more song-like than the *novel* word lists, *F*(3, 99) = 2.80, *p* = .044. Bonferroni-corrected post hoc *t* tests show that the *novel* word lists in Block 1 (*M* = 2.61, *SD* = .77) were rated equivalently to the *novel* word lists in Block 4 (*M* = 2.76, *SD* = 1.01), *t*(33) = 1.003, *p* = 1.00, but the *familiar* word lists in Block 1 (*M* = 2.45, *SD* = .76) were rated less song-like than the *familiar* word lists in Block 4 (*M* = 3.06, *SD* = 1.09), *t*(33) = 3.74, *p* = .007. The size of the effect comparing *familiar* word lists in Blocks 1 and 4 was considered to be medium in magnitude (Cohen’s *d* =.65 as computed in Lenhard & Lenhard, 2016).

### Discussion

The results of Experiment 1 show that word lists in the *familiar* condition that were presented several times throughout the course of the experimental session were rated as being more song-like at the end of the session than *novel* word lists that were only presented once during the experimental session. This finding provides empirical evidence that memory traces for previously presented word lists can influence the subsequent phenomenological experience of the speech-to-song illusion, as indicated by the increase in song-ratings to the same stimulus presented several times during the experimental session.

Observing the influence of memory on the subsequent phenomenological experience of the speech-to-song illusion in the present study is interesting, in part, because the stimulus used in the present study (i.e., word lists) was devoid of much of the acoustic, semantic, and syntactic information found in the phrases that are extracted from sentences, and that are used more often as stimuli to evoke the speech-to-song illusion (e.g., Deutsch et al., 2011). We predict that using “richer” stimuli containing additional features to encode in memory—such as phrases extracted from sentences—is likely to result in a larger effect than the effect size observed in the present study using more impoverished stimuli. We await future studies to confirm this prediction.

Demonstrating in a controlled laboratory setting that memory traces for previously presented word lists can influence perception of an illusion at a later point in time also lends some credence to the anecdotal reports that subsequent presentations of the phrase “sometimes behave so strangely” appear song-like more quickly, even if several hours or days have passed. We acknowledge that the present experiment only tested memory in a session that lasted approximately 10–15 minutes, and not over hours or days; testing with a longer delay would certainly strengthen the present findings. Nevertheless, the present result does suggest that memory traces may exert influences on the perception of the speech-to-song illusion.

The present finding also complements the findings of Gronveld et al. (2020) who manipulated the contour of the fundamental frequency of the speech segments to make the stimuli increasingly more or less likely to elicit the speech-to-song illusion. Recall that they found that only the manipulation from song-like to less-song-like resulted in listeners continuing to give higher overall song-like ratings in the experimental session, suggesting that listeners cannot “unhear” the initial memory trace of a canonical, song-like stimulus. That memory for the canonical, song-like stimulus influenced the ratings to subsequent presentations of the phrase even when the auditory signal in the subsequent presentations was less than optimal for eliciting the speech-to-song illusion.

In contrast to Gronveld et al. (2020), we did not manipulate the physical stimulus during the experimental session. Rather, word lists were presented either once or multiple times during the experimental session. Thus, not only do variations in the perceptual features of the physical stimulus influence how one experiences the speech-to-song illusion (e.g., Falk et al., 2014), but memory traces of previously experienced stimuli also influence perception of the speech-to-song illusion.

Our longstanding interest in spoken word recognition (e.g., Vitevitch & Luce, 1998) led us to examine the speech-to-song illusion through the lens of a language processing model, namely NST (Castro et al., 2018). Finding that memory traces of previously experienced stimuli can influence the subsequent perception of the speech-to-song illusion led us to wonder about the *nature* of the memory trace of the stimulus in the speech-to-song illusion, a question often examined in spoken word recognition research (e.g., Vitevitch et al., 2014; Vitevitch & Donoso, 2011). Specifically, are the representations exemplar-based or more abstract in nature? Given that most research on the speech-to-song illusion has been from the perspective of music cognition (e.g., Deutsch et a., 2011; Margulis & Simchy-Gross, 2016), we believe that asking about the nature of the memory trace highlights the importance and value of examining psychological phenomena like the speech-to-song illusion from multiple and different perspectives.

## Experiment 2

The store of words that one knows in a given language is referred to as the *mental lexicon.* In the area of spoken word recognition there has been much debate about whether the mental lexicon stores abstract or exemplar representations (e.g., Goldinger, 1996). Abstract representations are much like the nodes in NST, which represent idealized *linguistic* information, but strips away *indexical* information (e.g., age, gender, speech disorder) associated with a unique speaker. In contrast, exemplar representations contain both linguistic and indexical information for every word ever produced by any speaker one has heard. Given the influence of memory traces influencing subsequent perception of the speech-to-song illusion that was observed in Experiment 1, we sought in the present experiment to examine whether abstract or exemplar representations influenced the speech-to-song illusion.

In the original study of the speech-to-song illusion Deutsch et al. (2011) repeatedly presented (among other conditions) the phrase “sometimes behave so strangely” in an untransformed manner, or repeatedly presented the phrase with the syllables in the phrase in a “jumbled” order in each repetition. Despite the same words being presented and spoken by the same speaker (i.e., similar acoustic/speech information in both conditions), the speech-to-song illusion was only observed in the untransformed condition, suggesting that exemplar-based representations may drive the speech-to-song illusion. In contrast, Gronveld et al. (2020) found that the speech-to-song illusion could still be evoked (in one condition) even when the F0 contour was manipulated up to 90% across repetitions, suggesting that a more abstract, canonical representation may drive the speech-to-song illusion. Note that scrambling the order of words in a phrase and altering the pitch contour of a phrase are rather extreme acoustic manipulations to make to the stimulus. Thus, a more subtle manipulation may be required to discern if exemplar or abstract representations are involved in the speech-to-song illusion.

To test whether exemplar or abstract representations are involved in the speech-to-song illusion, we used a slightly more subtle manipulation that is commonly used to examine so-called specificity effects in spoken word recognition—namely, we presented stimuli produced by the same talker or by different talkers (e.g., McLennan & Luce, 2005). Switching talkers can be argued to be a subtle manipulation because previous studies have shown that listeners often fail to detect when changes in the talker occur, a phenomenon known as *change deafness* (Vitevitch, 2003).

In the present experiment, lists of words recurred during the experimental session with participants rating them on a 5-point scale, as in Experiment 1. In contrast to Experiment 1, all the word lists recurred throughout the experimental session, but this time the lists recurred in either the same voice or in a different voice.

If abstract representations influence the speech-to-song illusion, then, as in Experiment 1, we should see an increase in the song-likeness ratings for word lists that recur in the same voice and in a different voice. However, if exemplar representations influence the speech-to-song illusion, then we should see a main effect for the recurrence of the word lists (replicating Experiment 1), as well as an interaction such that the word lists presented again in the same voice will be rated as more song-like than the word lists that recur, but in a different voice later in the session.

### Methods

#### Participants

Based on the effect sizes, statistical power, and sample sizes of previous speech-to-song experiments (e.g., Castro et al., 2018), we established the stopping rule of collecting data from 40 participants or the end of the semester occurs, resulting in data being collected from 39 native English speakers recruited from a pool of students enrolled in Introductory Psychology at the University of Kansas by the end of the semester. Participants received partial credit toward the completion of the course for their participation. All were native English speakers, none reported a hearing or speech disorder, and none participated in Experiment 1. Written informed consent was obtained before participating in the experiment, and this experiment was approved by the institutional review board at the University of Kansas.

#### Stimuli

Because specificity effects can be subtle, we wished to maximize the likelihood of eliciting the speech-to-song illusion, so we used only the word lists with dense words used in Experiment 1 (originally recorded by a female speaker) in the present experiment. The same words were also recorded in the same manner by a male speaker (MSV) and edited in the same manner as the word lists used in Experiment 1. In order to use all the word lists, and to counterbalance the switch in voices, Lists 1-6 (referred to as Order A) were presented to half of the participants and Lists 2-7 (referred to as Order B) were presented to the remaining participants (see Table 2 for a visual representation of how the lists were presented to participants during the experimental session).

**Table 2:** A visual representation of how the stimuli (in Order B as designated in the Appendix) were presented in the experimental session

	Block 1	Block 2	Block 3	Block 4
Same voice	List-2-F	List-2-F	List-2-F	List-2-F
	List-3-M	List-3-M	List-3-M	List-3-M
Switched voice	List-4-F	List-4-F	List-4-M	List-4-M
	List-5-M	List-5-M	List-5-F	List-5-F
	List-6-M	List-6-M	List-6-F	List-6-F
	List-7-F	List-7-F	List-7-M	List-7-M

*Note*. Each participant received the same six word lists within each block, but presentation order within each block was in a different randomized order for each participant. F = female speaker. M = male speaker. If the voice of the talker changed (as in the last four lists) it occurred in Block 3 and remained that talker in Block 4

The six lists were presented four times each during the experiment, resulting in a total of 24 trials. These 24 trials were separated into four blocks. Each block contained one randomly ordered presentation of each of the six word lists. One of the lists was presented in the female voice in each block, and a different list was presented in the male voice in each block. These word lists will be referred to as the *same-voice* condition, and appear in the top two rows of Table 2. As in Experiment 1, there was no time delay between the presentation of each block; we simply use the term “block” to facilitate description of how the stimuli were presented during the experimental session.

Two word lists were presented in the female voice in Blocks 1 and 2, and then in the male voice in Blocks 3 and 4. The remaining two word lists were presented in the male voice in Blocks 1 and 2, and then in the female voice in Blocks 3 and 4. These word lists (in the bottom four rows of Table 2) will be referred to as the *switched-voice* condition. As in Experiment 1, the identical list of four words was repeated; there were not different tokens of each word, or any variation in the acoustics across the 10 repetitions of each list (see the Appendix for the words in each word list and for which word lists were presented in which voice).

#### Procedure

The same equipment and procedure used in Experiment 1 were used in the present experiment.

### Results

**Fig. 3:**
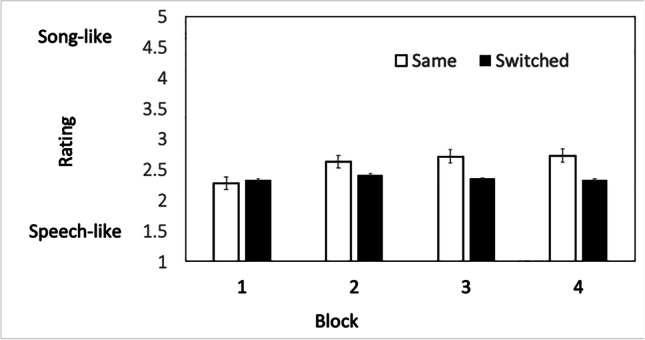
Mean song-like ratings (and standard error of the mean) in Experiment 2 for word lists that were repeated in the *same voice* across the four presentation blocks and word lists that were repeated, but the voice *switched* halfway through the experiment

A two-way (Blocks × Voice) repeated-measures ANOVA was used to analyze the data (see Fig. 3). There were four presentation blocks during the experimental session, and word lists were presented in the *same voice* throughout or the voice was *switched* (in Block 3). The main effect of block was significant, with word lists tending to be rated as more song-like with each presentation, *F*(3, 114) = 3.21, *p* = .03. The main effect of presentation voice was significant, with word lists presented in the *same voice* throughout the experiment being rated as more song-like than the word lists that were presented with *switched voices*, *F*(1, 38) = 10.65, *p* = .002.

Crucially, the interaction between blocks and voice was also statistically significant, suggesting that only the word lists that were repeated in the *same voice* throughout the experiment were rated as more song-like at the end of the experiment than the word lists that were repeated but had voices that *switched* halfway through the experiment, *F*(3, 99) = 2.81, *p* = .043. Bonferroni-corrected post hoc *t* tests confirmed that the word lists in Block 1 (*/M* = 2.33, *SD* = .73) were rated equivalently to the word lists in Block 4 (*M* = 2.34, *SD* = .61), *t*(33) = .045, *p* = 1.00, when the voice was *switched* halfway through the experiment. However, for word lists that were presented in the *same voice* throughout the experiment, the increase in song-like ratings from Block 1 (*M* = 2.28, *SD* = .86) to Block 4 was statistically significant (*M* = 2.73, *SD* = .83), *t*(33) = 3.65, *p* = .009. The size of the effect comparing word lists in the *same voice* in Blocks 1 and 4 was considered to be medium in magnitude (Cohen’s *d* =.53 as computed in Lenhard & Lenhard, 2016).

### Discussion

The results of Experiment 2 show that word lists repeated in the *same voice* throughout the experimental session were rated as being more song-like at the end of the session than word lists that were repeated during the experimental session, but in voices that *switched* halfway through the experimental session. As in Experiment 1, the present finding suggests that memory traces for previously presented word lists can influence subsequent ratings of the same stimulus appearing later in the experimental session.

More importantly, however, the results of the present experiment suggest that the memory traces that influence subsequent ratings in the speech-to-song illusion are not abstract in nature but are instead exemplar-based representations. Recall that evidence from Deutsch et al. (2011) using jumbled phrases suggested that exemplar representations might influence the speech-to-song illusion, whereas evidence from Gronveld et al. (2020) suggested that abstract representations might influence the speech-to-song illusion. Had we not viewed the speech-to-song illusion through the lens of spoken word recognition research and theories (e.g., NST), we would not have explored the nature of the memory traces (i.e., abstract vs. exemplar) that exert an influence on the perception of this illusion. Further, our use of a methodology commonly used in spoken word recognition research to examine voice-specificity effects (e.g., Nygaard & Pisoni, 1998) allowed us to demonstrate with a more subtle stimulus manipulation than employed by Deutsch et al. or Gronveld et al. that exemplar representations may also influence an auditory illusion in addition to spoken word recognition processes (Goldinger, 1996; see also Vitevitch & Donoso, 2011).

Evidence from McLennan and Luce (2005) suggests that abstract or exemplar lexical representations can be used during spoken word recognition depending on the effort of and time pressure on cognitive processing. For situations that require rapid processing or processing that is not effortful, abstract lexical representations are typically used. Only when processing is slow or effortful are exemplar representations employed. Thus, it is possible that both abstract and exemplar representations influence the perception of the speech-to-song illusion. Future studies of the speech-to-song illusion could perhaps employ speed/effort manipulations like those employed in McLennan and Luce (2005) in their studies of spoken word recognition to examine further the role of abstract and exemplar representations in the speech-to-song illusion. One way to manipulate processing effort might be to use meaningful phrases instead of the lists of words employed in the present experiment

Although most research on the speech-to-song illusion has examined the illusion from the perspective of music perception/cognition (e.g., Deutsch et a., 2011; Margulis & Simchy-Gross, 2016), the result from the present experiment highlights the importance and value of examining psychological phenomena like the speech-to-song illusion from multiple and different perspectives. Examining the speech-to-song illusion from the theoretical and methodological perspective of speech perception, spoken word recognition, and language processing may continue to provide novel insights into this unique auditory illusion. Indeed, the use of the language processing model, node structure theory (MacKay, 1987), to account for the speech-to-song illusion has already led to a number of important discoveries about this illusion (Castro et al., 2018; Mullin et al., 2021; Vitevitch et al., 2020). In the General Discussion, we compare the NST account of the speech-to-song illusion to other accounts of the speech-to-song illusion.

## General discussion

In the present two experiments we demonstrated that memory-traces of previously presented word lists can also influence perception of the speech-to-song illusion. Several accounts have been offered to explain the speech-to-song illusion. Simchy-Gross and Margulis (2018) suggested that “the speech-to-song illusion might depend on semantic satiation (Severance & Washburn, 1907) to suppress semantic associations before musical listening can emerge” (p. 4). Semantic satiation typically refers to the phenomenological experience of a speaker “losing” the meaning of a word that is produced overtly and repeatedly (see review by Esposito & Pelton, 1971), making it unclear how a phenomenon in speech production (i.e., semantic satiation) can be responsible for a phenomenon in speech perception (i.e., the speech-to-song illusion). Simchy-Gross and Margulis (2018) did not elaborate on how one phenomenon produces the other, nor on a common cognitive mechanism that might underlie both phenomena.

Note that more recent work on semantic satiation has shown that it can also occur with repeated visual or auditory presentation of words (Kounios et al., 2000), suggesting that semantic satiation could influence a perceptual phenomenon like the speech-to-song illusion. However, the semantic satiation account of the speech-to-song illusion is challenged by the fact that the illusion can be elicited with nonwords as well as words from a language that one does not know (e.g., Experiments 3 and 4 of Castro et al., 2018; Margulis et al., 2015). In the case of nonwords and words from a language that one does not know there are no semantic representations to satiate, making it unclear how semantic satiation could account for the speech-to-song illusion.

Another account of the speech-to-song illusion suggests that repetition of the stimulus causes the illusion (Margulis, 2013; Margulis & Simchy-Gross, 2016; Rowland et al., 2019). Clearly, repeated presentation of the stimulus to the listener is *necessary* for the illusion to occur, but repetition alone is not a *sufficient* explanation for how or why the illusion occurs. For example, why does repetition cause the percept to change from speech to song instead of to something else, such as other words or nonwords as occurs in another auditory illusion known as the verbal transformation effect (Warren & Gregory, 1958), in which a single word is presented repeatedly but appears to change to another word? One possibility is that repetition is more prevalent in music than it is in speech, perhaps accounting for why the percept switches to something song-like instead of some other form of speech (Margulis, 2013). However, that account fails to explain why the Verbal Transformation Effect, which also employs stimulus repetition, results in the percept changing from one word to another rather than to a song.

It is also not clear exactly what repetition is doing to cause a change in percepts from speech to song. Deutsch et al. (2011) hypothesized that

in listening to the normal flow of speech, the neural circuitry underlying pitch salience is somewhat inhibited, perhaps to enable the listener to focus more on other characteristics of the speech stream that are essential to meaning, i.e., consonants and vowels. We can also hypothesize that exact repetition of the phrase causes this circuitry to become disinhibited, with the result that the salience of the perceived pitches is enhanced. (p. 2251)

From a psycholinguistic perspective, the first hypothesis about what repetition is doing to cause a change in percepts is inconsistent with what is known about the languages of the world. Consider that variations in pitch is how tone languages, like Mandarin, convey meaning. (Recall that Mandarin was one of the other languages in which the speech-to-song illusion was observed; Zhang, 2011.) Similarly, pitch-accent languages like Japanese also rely heavily on pitch to convey meaning. Even stress-timed languages like English rely in part on variation in pitch to distinguish stressed from unstress syllables (e.g., CONtest vs. conTEST). It is unclear why the neural circuitry needed to process pitch, an essential component to understanding the meaning of words in the languages of the world, would be inhibited. It is also not clear what repetition is doing to this neural circuitry to then disinhibit it. Typically, repetition of a stimulus acts to habituate neural circuitry rather than activate it (e.g., Thompson & Spencer, 1966).

Finally, the repetition account proposed in Deutsch et al. (2011), in which pitch salience is so crucial, cannot explain how the speech-to-song illusion is elicited when there is little variation in pitch, as was the case with the lists of concatenated words used as stimuli in Castro et al. (2018), compared with the phrase “sometimes behave so strangely,” extracted from naturally produced speech used as a stimulus in Deutsch et al. (2011). Although the repetition account is commonly appealed to, it does not adequately explain how or why the speech-to-song illusion (as opposed to some other illusion) occurs in the first place, and why the phenomenological experience is the way that listeners report it. It is also unclear how memory affects the perception of the illusion in either the semantic satiation or repetition account of the speech-to-song illusion.

In the case of NST, the perception of speech occurs primarily via abstract representations (i.e., the nodes in Fig. 1 representing linguistic information, but not characteristics about individual speakers). However, NST does allow for learned experience/memory traces to influence processing (MacKay, 1987). For example, someone seeing the ambiguous duck–rabbit figure might first perceive it as a rabbit if they hear the word *carrot* and retrieve from memory the semantic relationship between carrots and rabbits. In contrast, another person viewing the same exact figure may hear *quack*, retrieve from memory the semantic relationship between ducks and the onomatopoeic noise they make, and instead perceive the ambiguous figure as a duck. The present results further demonstrate that learned experience/memory of listeners can indeed influence the perception of the speech-to-song illusion. Furthermore, the results of the present experiment suggest that some of those memory traces in NST may be exemplar based and can influence perception.

Looking at the speech-to-song illusion through the lens of spoken word recognition research and theories (e.g., NST) places the illusion into a rich theoretical context that allows us to explore this illusion in new ways and to connect it to a wide range of perceptual and cognitive phenomena, such as the influence that exemplar representations in the mental lexicon may have on various perceptual and cognitive processes. Further, NST has been used to account for a wide range of phenomena, including word retrieval and production (MacKay, 1987), tip-of-the-tongue states (Burke et al., 1991), differences in language processing due to aging (e.g., MacKay & Burke, 1990), the language production deficits of H.M. (MacKay et al., 1998), and another auditory illusion known as the verbal transformation effect (MacKay et al., 1993). In contrast, the repetition-based account of the speech-to-song illusion (Margulis, 2013; Margulis & Simchy-Gross, 2016; Rowland et al., 2019) appears ad hoc, and does not connect the illusion to other widely studied perceptual or cognitive phenomena.

The richer theoretical context afforded by the NST account also allows us to explore the illusion and its implications for music and language processing more broadly. Given that music is already used in many therapeutic interventions for speech and language disorders (e.g., Cohen, 1994), and work by Ma et al. (2020) demonstrated that song and infant-directed speech facilitates the process of word learning in adults, continued investigation of the speech-to-song illusion may increase our understanding of the perceptual and cognitive systems that underlie the illusion, and lead to the development of novel interventions for certain speech-related and language-related disorders. Continued investigation of the speech-to-song illusion may also increase our understanding of the relationship between language and music (e.g., Patel et al., 1998; Peretz et al., 2015; Tierney et al., 2021).

Given the well-known relationships between language and music (e.g., Jackendoff, 2009; Patel et al., 1998; Peretz et al., 2015; see also Layman & Dowling, 2018), there might be much value examining phenomena like the speech-to-song illusion from a perspective that combines language and music processing. The present set of experiments clearly highlights the value of looking at the speech-to-song illusion from the psycholinguistic perspective. However, NST has little to say about the representation of musical information or how it might affect language processing, which limits the extent to which this theory can account for other forms of auditory processing, including other auditory illusions. For example, Simchy-Gross and Margulis (2018) recently described the discovery of the sound to music illusion where the repetition of nonspeech sounds (e.g., ice cracking, shovel dragged across pavement) leads to increased ratings of music-likeness. It is unclear how the language processing model NST would account for the sound to music illusion.

Finally, the resurgence on social media of the brainstorm versus green-needle illusion (https://time.com/5873627/green-needle-brainstorm-explained/) suggests that the general public is interested in and entertained by perceptual illusions. Recent research suggests animals commonly found in zoos or other captive settings also have their environment enriched by certain perceptual illusions (Regaiolli et al., 2019), suggesting that the mechanisms responsible for certain perceptual illusions may have evolutionarily old origins. In addition to being entertaining to the general public (and zoo animals), perceptual illusions provide researchers with a way to examine the limits of the perceptual and cognitive systems involved in various illusions, thereby increasing our fundamental understanding of these systems, and making perceptual illusions worthy of further scientific investigation (Gregory, 1968; see also Boyette et al., 2020; McGuire et al., 2016; Vitevitch, 2003; Vitevitch & Donoso, 2011; Vitevitch et al., 2013; Vitevitch & Siew, 2017).

## Appendix

### Word lists used in Experiment 1

**Table:**

Dense Word lists				Sparse Word lists			
lever	battle	furry	candle^A^	lumber	badger	formal	cancer^A^
letter	muscle	berry	babble	lawyer	mother	button	barrel
polar	bubble	money	ladder^B^	person	beggar	movie	lucky^B^
cattle	banner	tackle	hurry	cashew	burden	tower	hero
leather	valley	puddle	candy	lady	vapor	powder	camel
dairy	meter	body	lighter	devil	mighty	bottom	lotion
paddle	shallow	mayor	worry	purple	shower	mitten	water

The superscripts (^A^ and ^B^) refer to the dense and sparse lists that were in the *familiar* condition for the two pseudo-randomized presentation orders. All other lists were in the *novel* condition and presented only once during the session

### Word lists used in Experiment 2

**Table:**

Dense Word lists				Order A	Order B
lever	battle	furry	candle	F-F	Not used
letter	muscle	berry	babble	M-M	F-F
polar	bubble	money	ladder	M-F	M-M
cattle	banner	tackle	hurry	M-F	F-M
leather	valley	puddle	candy	F-M	M-F
dairy	meter	body	lighter	F-M	M-F
paddle	shallow	mayor	worry	Not used	F-M

M-M, F-F, M-F, and F-M refer to the male (M) or female (F) voice. The M-M and F-F lists constituted the *same-voice* condition, whereas the F-M and M-F lists constituted the *switched-voice* condition
